# Virtual screening for inhibitors of the human TSLP:TSLPR interaction

**DOI:** 10.1038/s41598-017-17620-7

**Published:** 2017-12-08

**Authors:** Dries Van Rompaey, Kenneth Verstraete, Frank Peelman, Savvas N. Savvides, Koen Augustyns, Pieter Van Der Veken, Hans De Winter

**Affiliations:** 1 Laboratory of Medicinal Chemistry, University of Antwerp, 2610 Wilrijk, Belgium; 2 Laboratory for Protein Biochemistry and Biomolecular Engineering, Department of Biochemistry and Microbiology, Ghent University, 9000 Ghent, Belgium; 3 VIB-UGent Center for Inflammation Research, 9052 Zwijnaarde, Belgium; 4 VIB-UGent Center for Medical Biotechnology, 9000 Ghent, Belgium

## Abstract

The pro-inflammatory cytokine thymic stromal lymphopoietin (TSLP) plays a pivotal role in the pathophysiology of various allergy disorders that are mediated by type 2 helper T cell (Th2) responses, such as asthma and atopic dermatitis. TSLP forms a ternary complex with the TSLP receptor (TSLPR) and the interleukin-7-receptor subunit alpha (IL-7Rα), thereby activating a signaling cascade that culminates in the release of pro-inflammatory mediators. In this study, we conducted an *in silico* characterization of the TSLP:TSLPR complex to investigate the drugability of this complex. Two commercially available fragment libraries were screened computationally for possible inhibitors and a selection of fragments was subsequently tested *in vitro*. The screening setup consisted of two orthogonal assays measuring TSLP binding to TSLPR: a BLI-based assay and a biochemical assay based on a TSLP:alkaline phosphatase fusion protein. Four fragments pertaining to diverse chemical classes were identified to reduce TSLP:TSLPR complex formation to less than 75% in millimolar concentrations. We have used unbiased molecular dynamics simulations to develop a Markov state model that characterized the binding pathway of the most interesting compound. This work provides a proof-of-principle for use of fragments in the inhibition of TSLP:TSLPR complexation.

## Introduction

**Figure 1:**
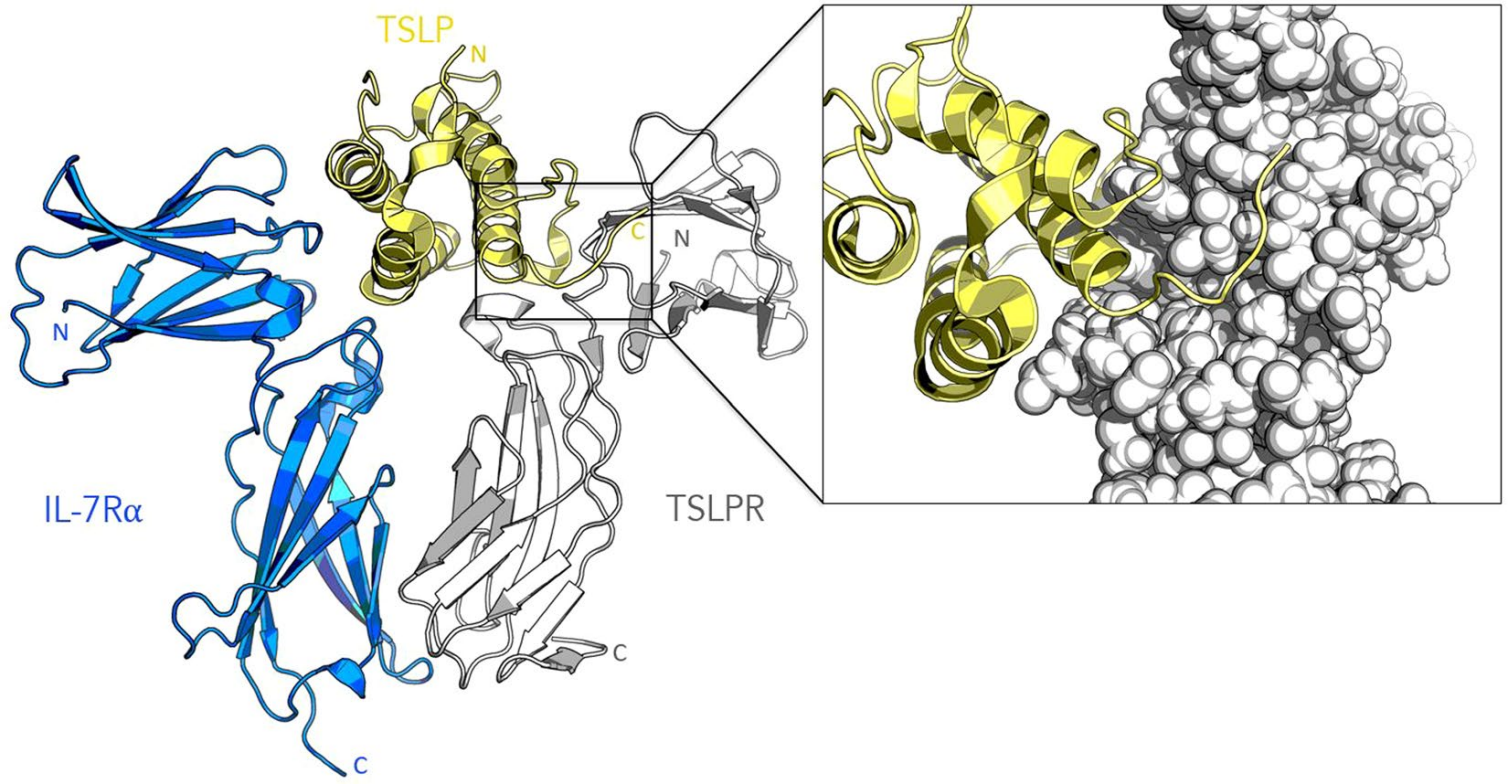
Cartoon representation of the human TSLP:TSLPR:IL-7Rα crystal structure. The inset shows a close-up of the TSLP:TSLPR interface, with TSLPR shown as spheres to illustrate the binding cleft. The figure is based on Protein Data Bank entry 5J11.

The pro-inflammatory cytokine, thymic stromal lymphopoietin (TSLP), is produced by skin keratocytes and epithelial cells of the lung and gut in response to pathogenic stimuli. TSLP signaling causes dimerization of the cognate TSLP receptor (TSLPR) and the interleukin-7-receptor subunit alpha (IL-7Rα) at the cell surface. TSLP is initially captured at the cell surface by a high affinity interaction with TSLPR (kD = 32 nM). Thereafter, IL-7Rα is recruited to the TSLP:TSLPR complex in a high affinity manner (kD = 29 nM) to elicit further intracellular signaling^1–3^. Figure 1 shows the ternary complex. This then drives the development of immature dendritic cells, mast cells, basophils, eosinophils and lymphocytes into a type 2 polarizing phenotype^4,5^, which in turn regulates immunity at these barrier surfaces. Dysregulation of this system is known to result in the development of atopic disease, which is a group comprised of three different conditions: asthma, atopic dermatitis and atopic rhinoconjunctivitis^6^. These diseases place a significant burden on healthcare systems throughout the world. In addition to its role in atopic disease, TSLP has also been implicated in various other pathophysiologies, such as chronic obstructive pulmonary disorder and corticosteroid resistance^5^. The potential role of TSLP in cancer development/progression is currently unclear and is still a topic of ongoing investigation. While some studies have provided evidence for a tumor-promoting role^7,8^, other studies have shown that TSLP does not play any significant role in tumor progression^9,10^.

The relevance of TSLP as a therapeutic target has been demonstrated in a clinical trial conducted by Amgen, where administration of the TSLP blocking antibody, Tezepelumab, resulted in a decrease of the baseline variables for airway inflammation, as well as a reduced sensitivity to various allergens^11,12^. TSLP traps, which are constructed by fusing the TSLPR and IL-7Rα extracellular regions together, have also demonstrated promising results^3,13^. Unfortunately, biologicals generally suffer from limited tissue penetration, a lack of oral bioavailability and high production costs^14^. Furthermore, they often present with complicated intellectual property situations^15^. We therefore feel a small molecule approach would provide a useful parallel form of targeting. Protein-protein interactions have generally been regarded to be difficult targets for the development of small molecule inhibitors, due to their oftentimes flat and extensive nature. However, it appears that not all residues contribute equally: a limited number of residues is often responsible for the majority of binding energy^16^. Residues accounting for more than 2 kcal/mol of the binding energy are known as hotspots. Bogan *et al*. found that tyrosine, tryptophan and arginine residues are over-represented in hotspots^17^. The discovery of hotspots has been of critical importance for the field of small molecule protein-protein inhibitors and as such, inhibitors for various targets have been developed in recent years, for instance Amgen’s MDM2-p53 inhibitor AMG232^18^, Sunesis Pharmaceuticals’ IL2-IL2R inhibitor SP4206^19^ or AbbVie’s ABT-199^20^, which targets B-cell lymphoma-2 (BCL2). In order to provide sufficient binding energy to compete with the native protein-protein interaction, protein-protein inhibitors tend to span several small pockets, making them larger than conventional drug molecules^21^. The aim of this study was to analyze the TSLP:TSLPR:IL7Rα complex with an emphasis on the druggability and to leverage this information in order to identify a fragment capable of disrupting the formation of the complex through a combined virtual screening – *in vitro* screening cascade.

## Results

### Characterization of the TSLP:TSLPR interface

The crystal structure of the human TSLP:TSLPR:IL-7Rα complex has recently been elucidated through X-ray crystallography by Verstraete and coworkers (PDB code 5J11)^12^. The TSLPR-binding epitope of TSLP consists of the TSLP helix D, the C-terminus and a large section of the TSLP AB loop. TSLP and the TSLPR ectodomain demonstrate a pronounced electrostatic complementarity, with TSLP presenting a positively charged surface patch to the negatively charged TSLPR interdomain elbow. We elected to target the TSLP:TSLPR interface for three important reasons. Firstly, the binding of TSLP to TSLPR is a mechanistic prerequisite for the formation of the cooperative ternary complex^12,22^. In absence of TSLPR, IL-7Rα does not have any measurable affinity for TSLP. Secondly, through targeting TSLPR, we hope to avoid any off-target effects on the IL-7 pathway. Thirdly, the TSLP:TSLPR interface appears to be more tractable than the TSLP:IL7Rα interface from a drug-design context, due to the presence of a well-defined binding cleft and the polar character of the interface. The interface is shown in the Fig. 1 inset.

### Hotspot identification

**Figure 2:**
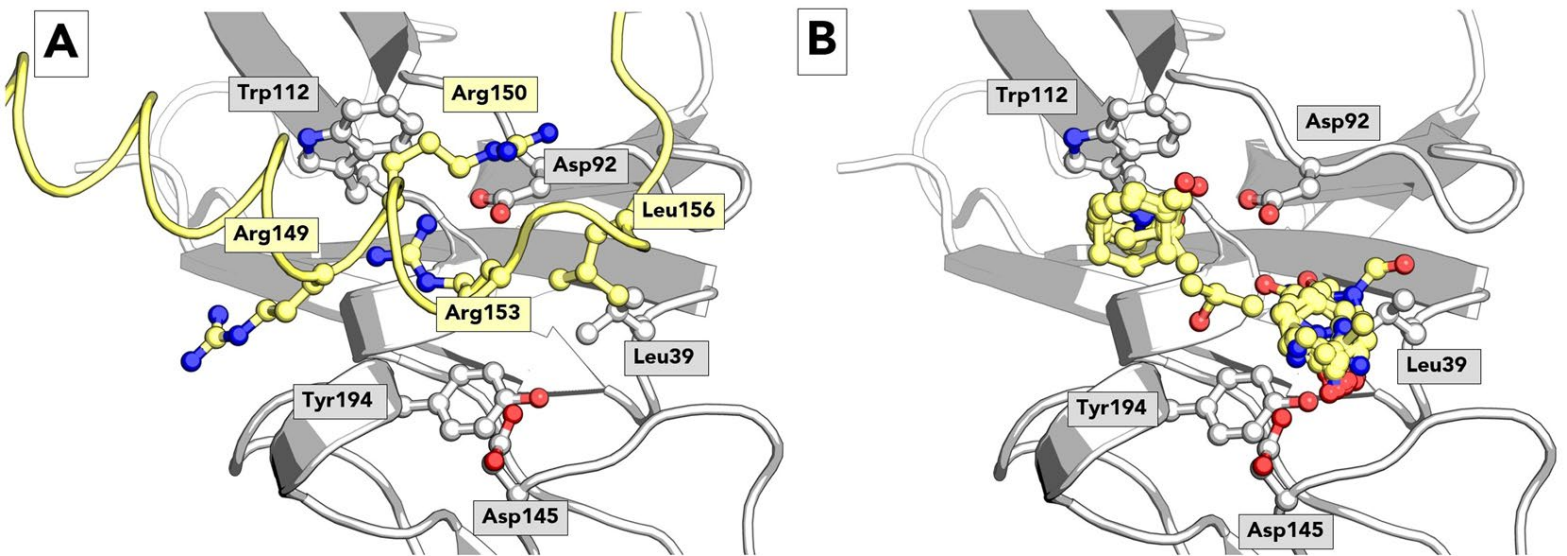
Hotspot analysis of human TSLPR. Panel A shows the TSLP:TSLPR interface as observed in the TSLP:TSLPR: IL-7Rα crystal structure. TSLP is shown in yellow cartoon representation, TSLPR in grey. Selected protein residues are shown in ball-and-stick, among which hotspot residues Arg149, Arg150 and Arg153 as identified by PredHS. Panel B shows FTMap probes in yellow ball-and-stick representation on the TSLPR surface. The two clusters are composed of the probes isobutanol, cyclohexane, dimethyl ether, ethanol, phenol, isopropanol, acetonitrile, methylamine, N,N-dimethylformamide, acetaldehyde, acetamide, ethane, acetone, urea and benzene.

We used established *in silico* methods to examine the TSLP:TSLPR binding site and evaluate possible hotspots. Possible hotspots in the TSLP:TSLPR complex were examined with PredHS, a machine learning web-based tool which predicts residues that account for the majority of binding energy based on structural neighborhood properties to identify hotspots in the TSLP:TSLPR complex^23^. The PredHS support vector machine method predicts TSLP residues TSLP-Arg149, TSLP-Arg150 and TSLP-Arg153 to be essential for the binding process as shown in Fig. 2A. This has been corroborated by mutagenesis results, where mutation of these residues has been shown to result in an increase in EC_50_^12^. Moreover, mutation of the contacting TSLPR residues TSLPR-Asp92 and TSLPR-Trp112 into alanine resulted in a thousand fold increase in EC_50_^12^. The three essential TSLP arginine residues bind to TSLPR in the binding cleft in which TSLPR-Asp92 and TSLPR-Trp112 are located, which is a promising result for the development of small molecule inhibitors.

As an orthogonal technique, computational solvent mapping was performed on TSLPR with FTMap^24^. This technique scans the surface of TSLPR using a variety of computational solvent probes, clustering the solvent probes to identify sites that are prone to binding small molecules. Two sites were identified near the TSLP binding cleft as displayed in Fig. 2B. The first site corresponds to the binding site of TSLP-Arg153, while the second site corresponds to the binding site of TSLP-Leu156. The agreement between these two techniques is an encouraging result for the development of TSLPR inhibitors.

### Docking of fragments to TSLPR

Based on our characterization of the binding interface, a 2400 Å^3^ search box was defined around the binding cleft. The 1,000 compound Enamine Gold Fragment library and the 2,500 compound Maybridge rule-of-three diversity library were then docked into TSLPR. Docking was performed using Autodock Vina 1.1.2^25^. The top five poses of each ligand were rescored using DSX 0.88^26^. The top scoring ligands, judged by raw scores as well as normalized scores, were inspected manually with specific attention to their conformations and the interactions with the pocket. Sixty fragments were selected for *in vitro* analysis and therefore purchased from their respective vendors (See Table S1 in the supplementary information for a complete list of fragments purchased with their respective scores).

### Biochemical evaluation

**Figure 3:**
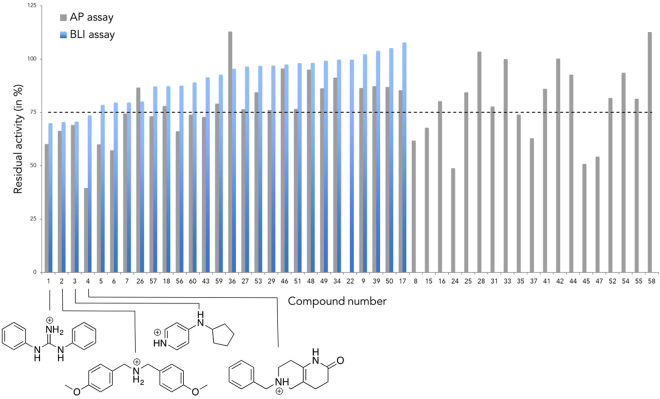
Assay inhibition data. The AP assay is shown in grey and the BLI assay is shown in blue. The percentages for each assay refer to the residual TSLP:TSLPR complex formation, i.e. strong inhibition of the complex resulted in a low residual percentage. The activity data for the four most active fragments is annotated with the chemical structure.

A two-stage biological screening was used to assess the *in vitro* activity of the fragments on the TSLP:TSLPR interface. Two different assays were performed to measure their inhibitory activity on the TSLP:TSLPR interface. The first assay was based on a TSLP-alkaline phosphatase fusion protein (TSLP-AP)^12^. TSLPR was incubated with TSLP-AP and the fragment at a concentration of 2.5 mM, followed by a washing step and quantification of the phosphatase activity of the fusion protein. This AP activity is a measure for TSLP:TSLPR binding, i.e. reduction of AP activity indicates reduced TSLP:TSLPR complexation. Eighteen fragments displayed less than 75% residual activity in the AP-based assay. The second assay employed bio-layer interferometry (BLI)^27^, whereby TSLP was immobilized onto a sensor tip and dipped into a solution containing TSLPR and a fragment at a concentration of 2.5 mM. The assay results for BLI and AP are shown in Fig. 3. Assay results for the BLI or AP assays are omitted for compounds with solubility issues under assay conditions. BLI results are omitted for compounds that result in aberrant traces.

Four fragments reduced the TSLP:TSLPR binding to less than 75% in both assays. Their structures are annotated in Fig. 3. We have opted to only pursue compounds that display sufficient inhibition in both assays, as activity in two or more orthogonal assays is an indicative of well-behaved fragments. Interestingly, while only 56% of purchased fragments have a net positive charge, all four of the top fragments are positively charged, consistent with the cationic nature of the natural ligand TSLP.

**Figure 4:**
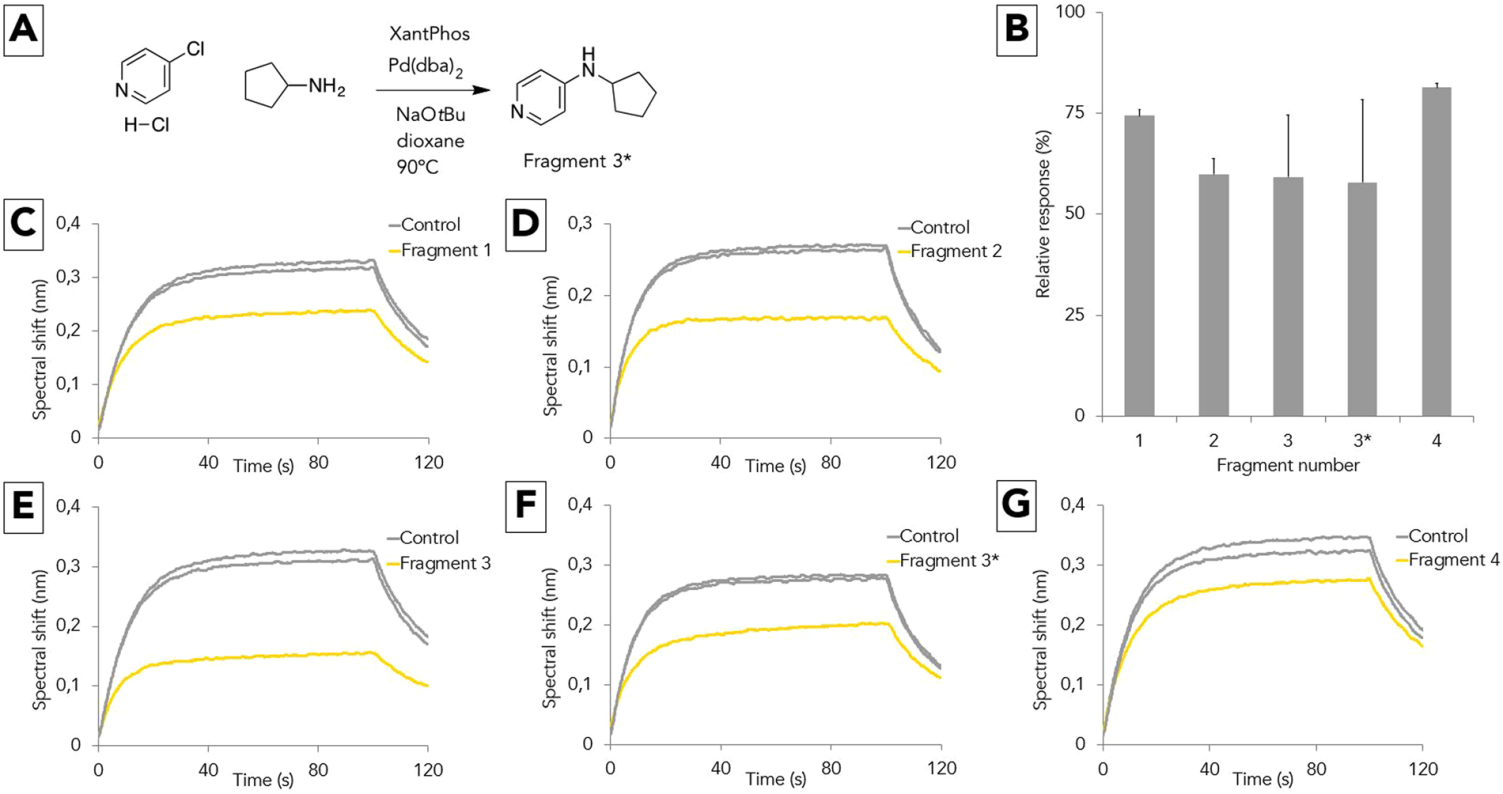
Synthesis and confirmatory BLI screening. Panel A shows the synthesis of fragment 3*. Panel B displays the average relative response of an *in duplo* BLI assay measuring the residual TSLP:TSLPR complexation for each of the fragments. Panels C–G show one representative BLI trace for each fragment.

Fragment 3 was selected for further investigation, as it is easily accessible through a Buchwald-Hartwig amination and its two constituent moieties, as well as analogues with varying substitution patterns, are readily available from commercial vendors. To confirm our initial results indicating that fragment 3 is a possible inhibitor of the TSLP:TSLPR interaction, it was resynthesized using literature protocols as illustrated in Fig. 4A^28^. This resynthesized compound will be denoted as fragment 3*. A second BLI screening was then performed in duplicate for the top four compounds and the resynthesized compound, confirming that fragment 3* exhibits similar inhibition to the original fragment 3 (Fig. 4B). One representative BLI trace is shown for each of the fragments in Figs. 4C–G.

The shallowness of the TSLPR groove and small size of the ligand renders assessment of the binding mode of this fragment through docking challenging. Several binding modes were considered to be plausible, and in order to identify the dominant binding mode, a high-throughput molecular dynamics experiment was conducted.

### Identification of the binding pathway

**Figure 5:**
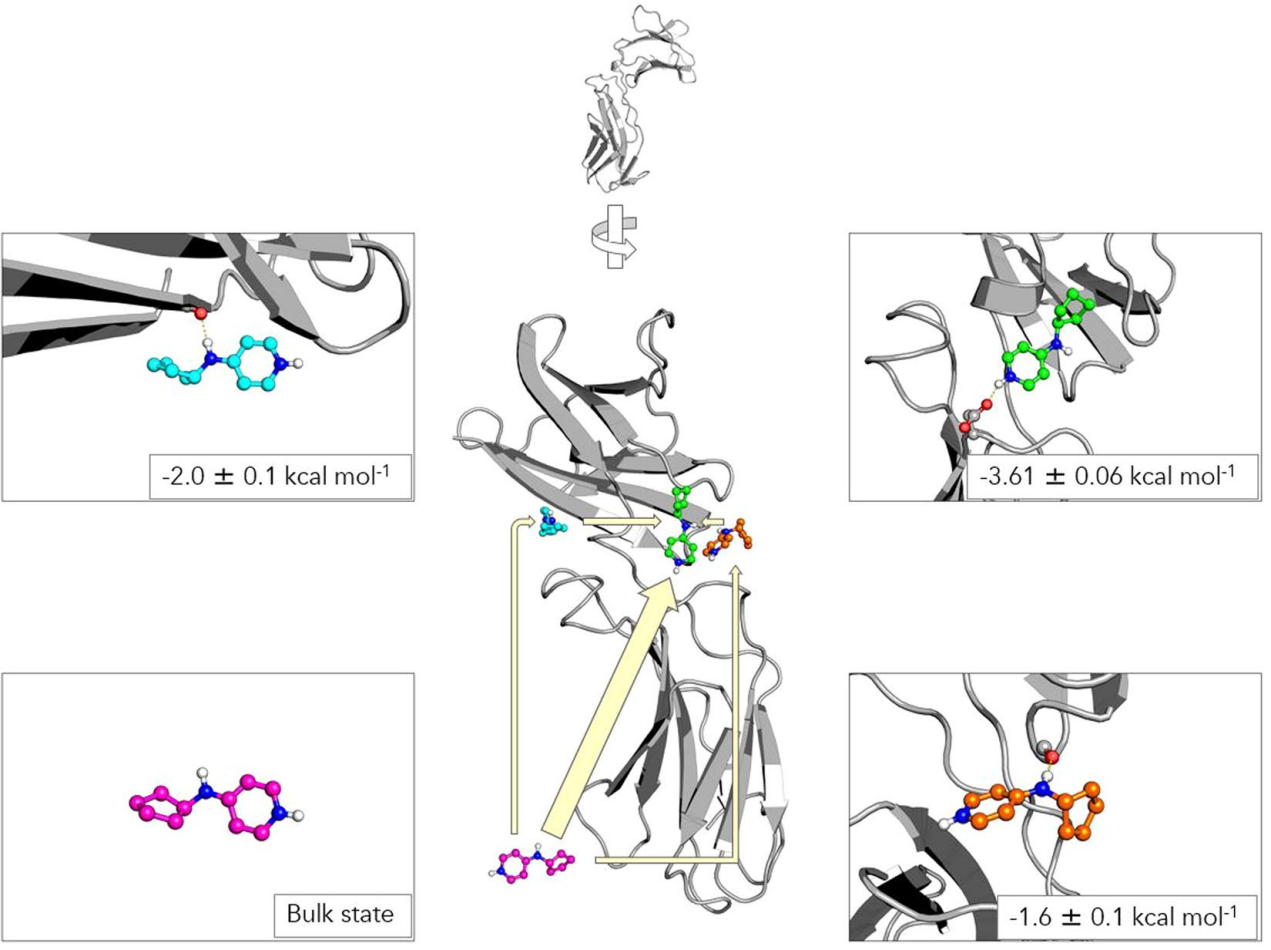
Four macrostates in a Markov state model for the binding of fragment 3 to human TSLPR. A single structure originating from the most representative microstate is shown for each macrostate. Binding energies predicted by the MSM are shown for each macrostate. The bulk state is shown in pink, the first metastable state in blue, the second metastable state in orange and the bound state is shown in green.

The underlying aim of this simulation was to simulate the binding process of the fragment to TSLPR. Our simulation setup consisted of TSLPR solvated in a water box, along with a randomly placed copy of fragment 3. No assumptions were made regarding the mode of binding: the ligand was placed in a random position and it was allowed to diffuse freely without any biasing potentials. A total of 150 molecular dynamics simulations of 250 ns each were performed using Gromacs^29^, for an aggregate sampling time of 37.5 microseconds. While this wealth of data is difficult to interpret manually, Markov state modelling may be used to generate a coarse-grained, easy to understand model of the binding process. Using the HTMD package, the Markov state model (MSM) was constructed as follows^30,31^. Contacts between the protein and ligand were used as features. These features were transformed using time-structure independent components analysis (TICA)^32^ with a lag time of 10 ns, retaining three dimensions and clustered into approximately 500 microstates with MiniBatchKMeans. These microstates were then used to construct an MSM, which resolved four kinetically distinct macrostates: the bulk state, two metastable states and the bound state (Fig. 5). A single structure originating from the most representative microstate is shown for each macrostate for clarity. Figure S1 shows twenty samples taken from each macrostate. The bulk state is defined by the ligand not interacting with the protein or with transient, unstable contacts. In the first metastable state, the ligand binds to a distal site of TSLPR, with occasional hydrogen bonding of the linker nitrogen to the Ile32 backbone carbonyl. In the second metastable state, the ligand binds near the TSLP binding site, with occasional hydrogen binding of the linker nitrogen to the Asn38 backbone carbonyl.

**Figure 6:**
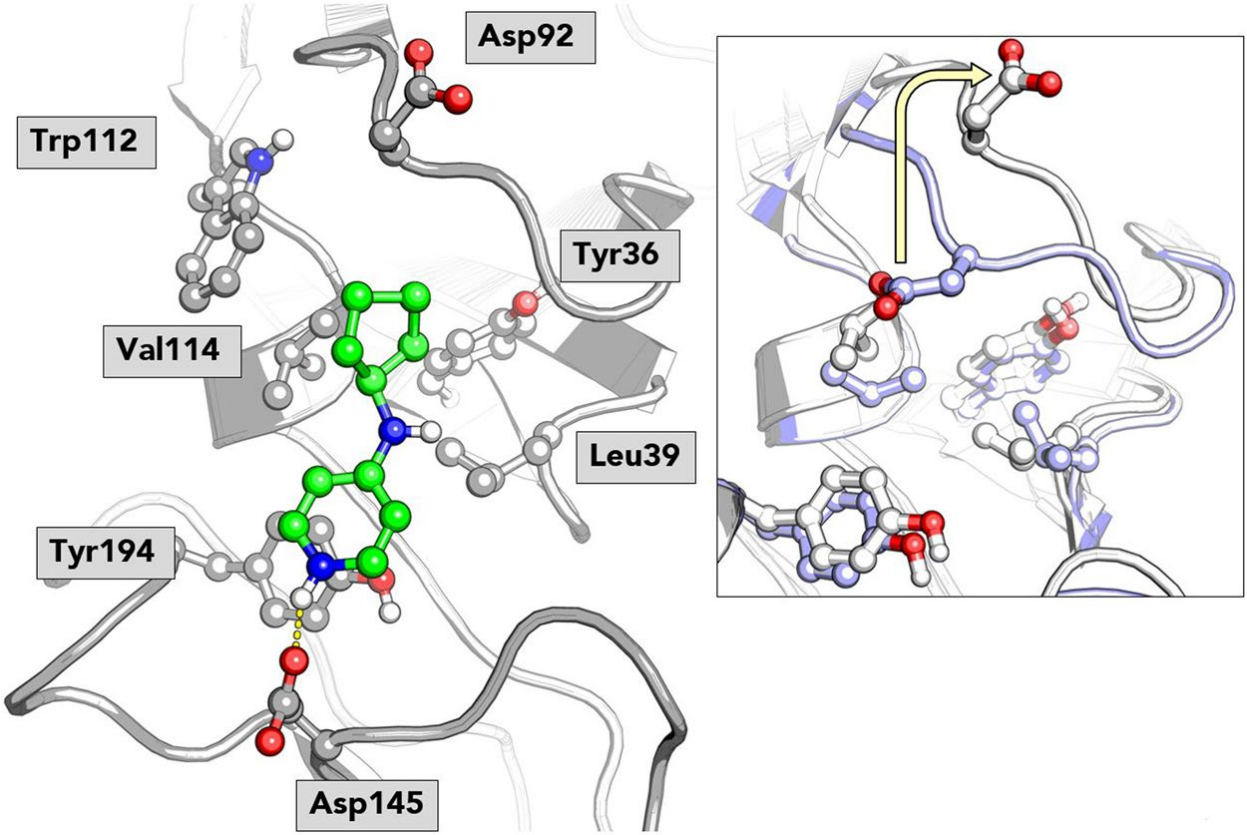
Proposed binding mode of fragment 3 as predicted by the MSM. The fragment is shown in green ball-and-stick representation. TSLPR is shown in grey cartoon with key residues shown in ball-and-stick. The inset shows an overlay of the TSLPR structure from our Markov state model (in grey) with the crystal structure (in violet), illustrating the lid-like opening of the loop containing Asp92.

Inspection of the most representative microstate of the proposed bound state reveals several key interactions as shown in Fig. 6. The positively charged pyridine nitrogen engages in a hydrogen bond interaction with Asp145, while the pyridine interacts with Tyr194 through a pi-stacking interaction. The cyclopentanyl moiety interacts with a lipophilic pocket formed by Tyr36, Leu39, Trp112 and Val114. An interesting conformational change can be observed in the macrostate: compared to the TSLP:TSLPR:IL7Rα crystal structure, the loop containing Asp92 opens up slightly in a lid-like manner, positioning Asp92 outwards as illustrated in the Fig. 6 inset. This conformation can also be observed in previous simulations of apo-TSLPR in water, prompting us to hypothesize that this may be more representative of the native TSLPR state than the conformation of Asp92 as observed in the crystal structure^12^. Furthermore, the cyclopentyl moiety of fragment 3 binds in a cavity which is partially occluded by Asp92 in the TSLP:TSLRP structure, indicating that the flip of the loop may thus be required for it to bind. This observation also has profound implications for docking studies targeting TSLPR, as the conformational change of Asp92 strongly affects the electrostatics of the binding site.

The MSM can elucidate the path followed by the ligand from the unbound bulk state to the bound state. In this case, the large majority of binding events occur through direct diffusion from bulk solvent (98.8%). Binding events where the ligand passes through the intermediate states were rare: 0.3% of binding events occur through the first metastable state, while the second metastable state accounts for 0.8% of binding events. Finally, the MSM can be used to estimate thermodynamics of binding. The dissociation constant K_D_ and the binding energy ΔG were calculated to be 1.9 ± 0.3 mM and −3.61 ± 0.06 kcal/mol, respectively, indicative of a relatively weak affinity fragment. This is consistent with our biochemical assays, in which high concentrations of ligand were required to elicit a reduction in TSLP:TSLPR complex formation. In future studies, we will aim to use the structural information obtained here for the development of this fragment into a lead-sized inhibitor.

## Discussion

The aim of our study was to explore a parallel avenue towards the development of TSLP:TSLPR interaction inhibitors. Indeed, biologicals have been the main paradigm in inhibition of TSLP signaling and in inhibition of protein-protein interfaces in general. One of the most prominent achievements in the field has been Amgen’s development of an anti-TSLP monoclonal antibody Tezepelumab, also known as AMG157. Currently in phase II clinical trials, it has been shown to be a useful tool for the management of moderate-to-severe asthma, highlighting the relevance of TSLP signaling as a therapeutic target. A fusion protein consisting of the ectodomains of TSLPR and IL-7Ra, also known as a TSLP-trap, has been developed by Verstraete *et al*. and was shown to inhibit TSLP-driven activation of dendritic cells^12^. Furthermore, a recent study by Park and coworkers has explored the potential of peptide-derived inhibitors, resulting in three peptides capable of TSLP inhibition at submillimolar concentrations^33^. However, biologicals generally present with a number of challenges: they have low tissue penetration, are not orally active and are expensive to produce. We therefore feel the development of a small molecule inhibitor would be a useful parallel avenue to explore.

In this study, we present the results of a proof-of-principle fragment–based screening campaign with the aim of identifying fragments capable of disrupting the TSLP:TSLPR complex. The interactions leading to this binary complex are a mechanistic prerequisite for the recruitment of the shared receptor IL-7Rα to establish the pro-inflammatory signaling assembly mediated by TSLP in allergy and asthma^3^. Following an in silico characterization of the TSLP:TSLPR interface, a docking experiment was performed to identify inhibitors. A set of sixty fragments was purchased in order to validate our results *in vitro*, of which four fragments were shown to be active in two different assays. A limitation of the assay setup is that both assays measure TSLP:TSLPR interaction, rather than directly quantifying the binding of the fragment to TSLPR. One fragment was selected for further examination due to its favorable structure and synthetic accessibility. We performed molecular dynamics in order to develop a Markov state model, which was used to explore the binding pathway and mode of binding. Our model indicates that the fragment binds at the TSLP binding site and that the TSLPR site undergoes a conformational change of the TSLPR site that may be required for fragment binding.

Going forward, the main challenge will be expanding the fragments identified in this work into more potent lead molecules. Such inhibitors could further help elucidate the potential role of TSLP in various pathophysiologies and could also be a starting point for the development of drugs targeting this pathway. Three main strategies for the elaboration of fragments have been used in literature: linking fragments that bind to different sites together chemically, merging fragments with existing scaffolds and growing fragments^34^. Fragment growing is the most commonly employed technique and will be the initial focus of follow-up research, as the binding sites of the fragments are not yet known, hindering the use of fragment linking, and no scaffolds for TSLPR inhibition have been described thus far, rendering fragment merging difficult. Crystallography of a fragment in complex TSLPR would be invaluable for fragment growing. Indeed, structural information about the complex under the form of crystal structures or NMR structures is regarded as indispensable for fragment optimization in some organizations. However, modeling techniques such as the MSM presented herein can also be used to guide the synthetic effort.

In conclusion, we have successfully used a combined virtual – *in vitro* screening approach to identify the first fragments to inhibit the TSLP:TSLPR interaction, providing a proof-of-principle for the use of fragments in the modulation of TSLP signaling.

## Materials and Methods

The coordinates for the crystal structure of the human TSLP:TSLPR:IL-7Rα complex used in all calculations were kindly provided by Kenneth Verstraete and a further refined version of this structure was deposited to the PDB under identifier 5J11^12^. PredHS hotspot prediction was performed on the TSLP:TSLPR complex. FTMap was run on TSLPR with the complex partners deleted in protein-protein interaction mode using the default probe set consisting of acetamide, acetonitrile, acetone, acetaldehyde, methylamine, benzaldehyde, benzene, isobutanol, cyclohexane, *N*,*N*-dimethylformamide, dimethyl ether, ethanol, ethane, phenol, isopropanol and urea.

The Enamine golden fragment database (1,000 compounds) and Maybridge fragment library (2,500 compounds) were downloaded from their respective vendor sites. The compound files were imported into MOE and processed through the database wash protocol. This protocol assigns relevant protonation states and generates 3D coordinates. These were then converted to Autodock Vina’s PDBQT format. The receptor was processed using the MOE^35^ structure preparation tool and converted into a PDBQT file.

All molecular dynamics simulations were performed with Gromacs 5.1.2^29^. General Amber force field (GAFF)^36^ parameters for the ligand were obtained using FESetup^37^. The TIP3P water model was used for solvent and Amber99sb-ildn was used for the protein and ions^38^.

All frames of the trajectory were centered on the protein backbone atoms and aligned to a common reference frame. The contacts between the protein and the ligand were then extracted as features. These features were transformed using a 10 ns lag time TICA, retaining three dimensions. MiniBatchKMeans clustering with a cluster number of 500 was then performed, merging clusters containing fewer than five points. These clusters were used to construct a Markov state model with a lag time of 15 ns and 4 macrostates.

### AP assay

Black 96-well Maxisorp plates (Nunc) were coated with 0.25 µg/ml anti-penta-His antibody (Qiagen) in 50 mM sodium carbonate/bicarbonate buffer pH 10.6., followed by a 2 hour blocking with 1% bovine serum albumin in Dulbecco’s phosphate buffered saline (Gibco). Wells were washed three times with TBS-T buffer (0.1% Tween, 25 mM Tris, 148 mM NaCl, 2 mM KCl, pH 7.4). Wells were incubated for 2 hours with 1 µg/ml recombinant his-tagged ectodomain of the human TSLP receptor^12^ in TBS-T buffer and subsequently washed three times with TBS-T. Wells were incubated for 4 hours with TBS-T containing 2.5 mM fragments, 2.5% DMSO and 1/60 diluted cell culture supernatant containing recombinant hTSLP-alkaline phosphatase fusion protein^12^. Wells were washed three times with TBS-T buffer, and bound alkaline phosphatase activity was determined using the PhosphaLight kit (Tropix) and an Envision chemiluminescence counter (PerkinElmer). Luminescence signals of samples containing fragments were compared with luminescence of samples containing hTSLP fusion protein and 2.5% DMSO without fragment.

### BLI assay

BLI experiments were performed in PBS-buffer supplemented with 0.01% (w/v) BSA, 0.002% (v/v) Tween 20 and 2.5% DMSO, with an Octet RED96 instrument (FortéBio), operating at 25 °C. Streptavidin-coated sensor tips were functionalized with mammalian-derived biotinylated human TSLP^R127A/R130S^ (NP_149024.1; residue 1–159) carrying a C-terminal AVITAG at its C-terminus^12^ and quenched with a 10 µg ml^−1^ biotin solution. The human TSLPR ectodomain (NP_071431.2; residue 1–221) was produced from stable transfected HEK293S-TetR MGAT1^−/−^ cells was used as analyte at a concentration of 100 nM^12^. Compounds were included in the TSLPR containing wells to give a final concentration of 2.5 mM. The data was analyzed using FortéBio Data Analysis 9.0.0.4. For all measurements a column of non-functionalized sensors was used to enable parallel reference subtraction.

Sixty compounds were tested in the first BLI-screen using one column of sensor tips functionalized with 1 nm of biotinylated hTSLP and one column of reference tips. By using eleven serial cycles of a 200 s association phase followed by a 700 s dissociation phase a single sensor tip was used to measure multiple compounds. The relative response for each measurement was calculated as the response at the end of the association phase divided by the average response of control samples (100 nM TSLPR without compound).

In the second BLI-screen five compounds were tested using one column of sensor tips functionalized with 1 nm of biotinylated hTSLP and one column of reference tips. Each tip was used for 22 serial cycles consisting of a 100 s equilibration phase, a 100 s association phase, a 20 s dissociation phase and a 3 × 5 s regeneration cycle in 0.5 M H_2_SO_4_. The relative response (%) was calculated as the response at the end of the association phase divided by the average response of a 100 nM TSLPR measured in the cycles directly preceding and following each control (100 nM TSLPR) or sample (100 nM TSLPR + compound). Each compound was measured in duplicate.

### Synthesis of N-cyclopentylpyridin-4-amine, fragment 3

4-chloropyridine hydrochloride (100 mg, 0.667 mmol), sodium 2-methylpropan-2-olate (250 mg, 2.60 mmol) and 9,9-dimethyl-4,5-bis(diphenylphosphino)xanthene (17.36 mg, 0.030 mmol) were placed in an argon flushed high pressure tube and suspended in dioxane (5 ml) which was previously degassed by bubbling with argon. This suspension was degassed by bubbling with argon. Tris(dibenzylideneacetone)dipalladium(0) (18.31 mg, 0.020 mmol) was added, after which the suspension was again degassed by bubbling with argon. Cyclopentanamine (0.071 ml, 0.667 mmol) was added and the suspension was degassed by bubbling with argon for 10 minutes. The tube was sealed and the reaction was stirred at 90 °C overnight. The resulting suspension was filtered through celite and washed with methanol. Reverse phase chromatography using a methanol-water gradient yielded N-cyclopentylpyridin-4-amine (67,4 mg, 0.413 mmol, 62% yield).

Proton NMR: 1H NMR (300 MHz, DMSO) δ 1.42 (2H, m), 1.55 (2H, m), 1.65 (2H, m) 1.91 (2H, m), 3.72 (1H, dd), 6.44 (2H, dd), 6.47 (1H, d), 7.98 (2H, dd).

### Data availability

The datasets generated during and/or analysed during the current study are available from the corresponding author on reasonable request.

## Electronic supplementary material

Supplementary information
