# Author Correction: Virtual screening for inhibitors of the human TSLP:TSLPR interaction

**DOI:** 10.1038/s41598-018-24798-x

**Published:** 2018-04-24

**Authors:** Dries Van Rompaey, Kenneth Verstraete, Frank Peelman, Savvas N. Savvides, Koen Augustyns, Pieter Van Der Veken, Hans De Winter

**Affiliations:** 1 Laboratory of Medicinal Chemistry, University of Antwerp, 2610 Wilrijk, Belgium; 2 Laboratory for Protein Biochemistry and Biomolecular Engineering, Department of Biochemistry and Microbiology, Ghent University, 9000 Ghent, Belgium; 3 VIB-UGent Center for Inflammation Research, 9052 Zwijnaarde, Belgium; 4 VIB-UGent Center for Medical Biotechnology, 9000 Ghent, Belgium

Correction to: *Scientific Reports* 10.1038/s41598-017-17620-7, published online 08 December 2017

The original version of this Article contained errors in the affiliation list. Frank Peelman was incorrectly listed as being affiliated with ‘VIB-UGent Center for Inflammation Research’. In addition, Affiliations 3 and 4 were inverted and the original Affiliation 3 incorrectly read ‘VIB Medical Biotechnology Center, 9000, Ghent, Belgium’ and now reads:

VIB-UGent Center for Medical Biotechnology, 9000, Ghent, Belgium.

These errors have now been corrected in the HTML and PDF versions of this Article.

