# Supplementary material for: Virtual screening for inhibitors of the human TSLP:TSLPR interaction
Source: Sci Rep. 2017 Dec 8;7:17211. doi: 10.1038/s41598-017-17620-7 (PMC5722893; doi:10.1038/s41598-017-17620-7)
Supplement: Supplementary file 1 — Supplementary information [file 41598_2017_17620_MOESM1_ESM.doc]

# Virtual screening for inhibitors of the human TSLP:TSLPR interaction

**Supplementary information**

Dries Van Rompaey, Kenneth Verstraete, Frank Peelman, Savvas N. Savvides, Koen Augustyns, Pieter Van Der Veken, Hans De Winter

| Number | Structure | Vina score | DSX score |
| --- | --- | --- | --- |
| 1 |  | -5.4 | -48.5170 |
| 2 |  | -5.4 | -67.181 |
| 3 |  | -4.4 | -48.1270 |
| 4 |  | -5.7 | -60.1760 |
| 5 |  | -4.6 | -65.744 |
| 6 |  | -4.5 | -59.683 |
| 7 |  | -4.6 | -66.685 |
| 8 |  | -5.7 | -66.018 |
| 9 |  | -4.2 | -32.325 |
| 10 |  | -5.8 | -72.043 |
| 11 |  | -5.1 | -73.477 |
| 12 |  | -4.3 | -58.016 |
| 13 |  | -5.6 | -64.205 |
| 14 |  | -5.4 | -68.318 |
| 15 |  | -5.1 | -60.707 |
| 16 |  | -4.8 | -57.013 |
| 17 |  | -4.5 | -38.382 |
| 18 |  | -5.4 | -64.399 |
| 19 |  | -6.2 | -62.093 |
| 20 |  | -5.8 | -72.021 |
| 21 |  | -5.8 | -66.632 |
| 22 |  | -5.7 | -58.017 |
| 23 |  | -5.8 | -71.217 |
| 24 |  | -4.7 | -61.036 |
| 25 |  | -5.0 | -59.937 |
| 26 |  | -5.0 | -54.385 |
| 27 |  | -4.7 | -53.959 |
| 28 |  | -5.7 | -56.068 |
| 29 |  | -3.2 | -40.036 |
| 30 |  | -5.9 | -66.952 |
| 31 |  | -5.4 | -66.480 |
| 32 |  | -5.4 | -62.650 |
| 33 |  | -3.5 | -37.169 |
| 34 |  | -4.5 | -66.419 |
| 35 |  | -4.4 | -62.812 |
| 36 |  | -5.7 | -60.978 |
| 37 |  | -5.1 | -61.004 |
| 38 |  | -5.3 | -57.305 |
| 39 |  | -4.1 | -37.365 |
| 40 |  | -4.3 | -51.111 |
| 41 |  | -5.5 | -64.356 |
| 42 |  | -5.8 | -58.842 |
| 43 |  | -3.7 | -46.464 |
| 44 |  | -5.5 | -59.578 |
| 45 |  | -4.6 | -67.115 |
| 46 |  | -5.7 | -60.897 |
| 47 |  | -5.3 | -63.798 |
| 48 |  | -4.2 | -40.334 |
| 49 |  | -6.0 | -79.822 |
| 50 |  | -6.0 | -71.311 |
| 51 |  | -4.3 | -49.343 |
| 52 |  | -3.4 | -38.240 |
| 53 |  | -5.3 | -67.440 |
| 54 |  | -5.7 | -66.007 |
| 55 |  | -5.9 | -64.901 |
| 56 |  | -4.5 | -54.215 |
| 57 |  | -5.4 | -60.635 |
| 58 |  | -5.5 | -66.799 |
| 59 |  | -4.3 | -48.414 |
| 60 |  | -4.7 | -51.076 |

Table S1: List of all purchased fragments annotated with their respective Autodock Vina and DSX scores.

**
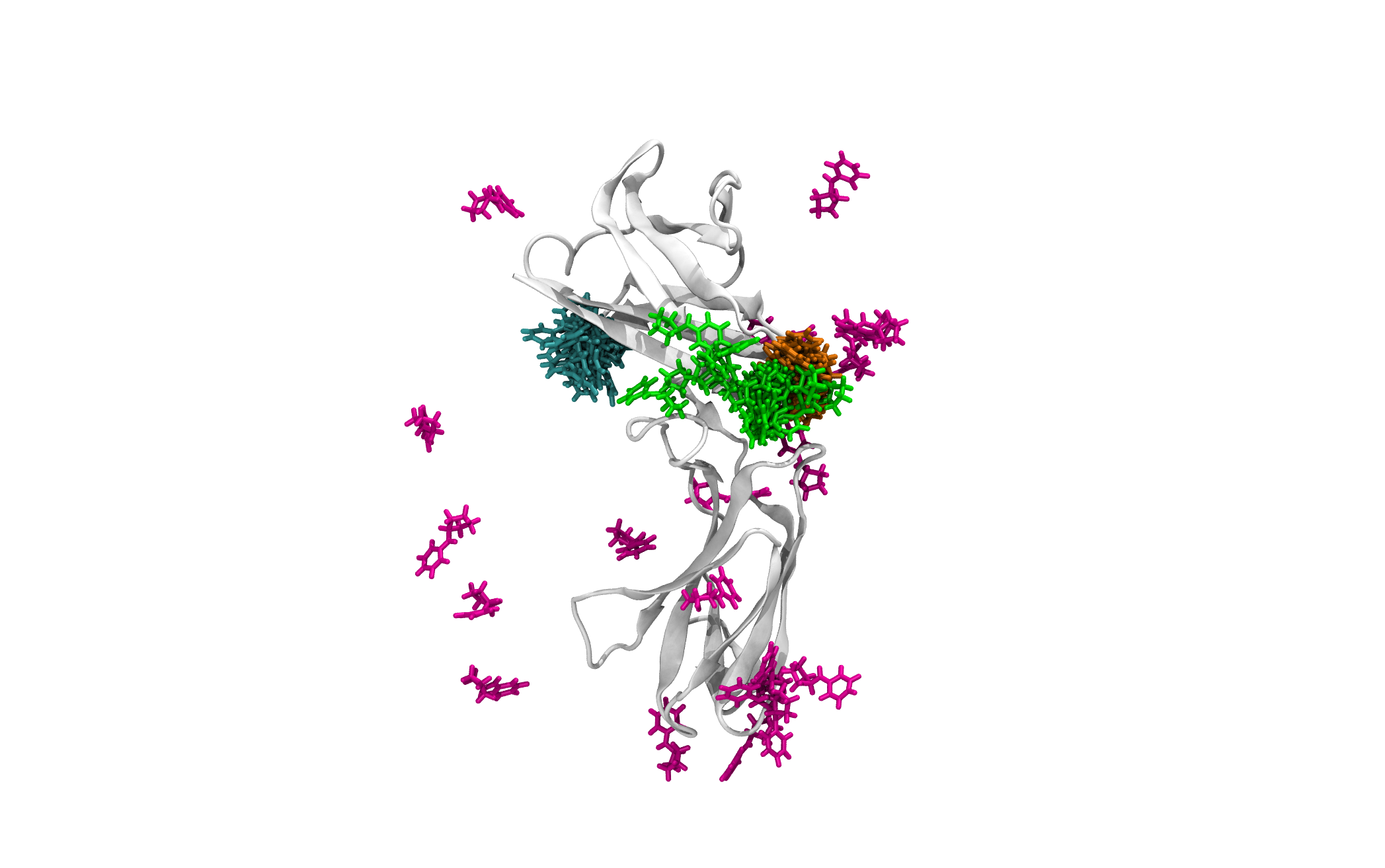
**

Figure S1: Four macrostates in a Markov State Model for human TSLPR. Twenty samples originating from each macrostate are shown. The bulk state is shown in pink, the first metastable state in blue, the second metastable state in orange and the bound state is shown in green.
